# Acute heart failure after Orthotopic liver transplantation: a case series from one center

**DOI:** 10.1186/s12871-018-0560-2

**Published:** 2018-07-31

**Authors:** Sonal Sharma, Kunal Karamchandani, Ryan Wilson, Sean Baskin, Dmitri Bezinover

**Affiliations:** 1 Department of Anesthesiology and Perioperative Medicine, Penn State College of Medicine/ Penn State Health Milton S. Hershey Medical Center, 500 University Drive, H187, P.O. Box 850, Hershey, PA 17033-0850 USA; 2 Department of Cardiology, Penn State College of Medicine/ Penn State Health Milton S. Hershey Medical Center, 500 University Drive, Hershey, PA 17033 USA

**Keywords:** Cirrhotic cardiomyopathy, Heart failure, Liver transplantation

## Abstract

**Background:**

Patients undergoing liver transplantation (LT) can develop acute heart failure (HF) in the postoperative period despite having had a normal cardiac evaluation prior to surgery. End-stage liver disease is often associated with underlying cardiac dysfunction which, while not identified during preoperative testing, manifests itself during or immediately after surgery.

**Case presentation:**

We describe three cases of non-ischemic acute HF developing shortly after LT in patients who had a normal preoperative cardiac evaluation. The challenges associated with both diagnosis and management of acute HF in the setting of a newly implanted graft will be discussed.

**Conclusions:**

Diastolic dysfunction, QTc interval prolongation, and an increase in BNP may be predictive of postoperative HF. Current recommendations for preoperative cardiovascular evaluation of transplant candidates does not include studies examining these risk factors and should be revised. Further investigations are necessary to evaluate these findings.

## Background

Acute heart failure (HF) after liver transplantation (LT) is associated with significant morbidity and mortality [1]. The etiology of HF in these patients is poorly understood. Despite extensive pre-operative cardiac evaluation, patients with end stage liver disease (ESLD) can have underlying cardiac dysfunction which is not identified during preoperative cardiac testing. There are a number of specific cardiac conditions associated with ESLD that can lead to acute cardiac deterioration in the immediate postoperative period.

We report a series of three cases of acute HF after LT. Left ventricular ejection fraction (EF) was normal in all three patients before transplantation but deteriorated dramatically in the immediate postoperative period after an uneventful surgical procedure. Our experience with these cases emphasizes the ambiguity associated with normal preoperative cardiac testing in LT candidates. Perioperative cardiac dysfunction specifically related to LT should be the subject of intensive investigation.

## Case descriptions

In all cases presented, patients received standardized intraoperative anesthetic management consistent with institutional protocol. After establishing noninvasive monitoring, general anesthesia was induced with propofol and fentanyl. Cisatracurium was used for muscle relaxation. After tracheal intubation, anesthesia was maintained with sevoflurane and fentanyl, and a continuous infusion of cisatracurium.

In addition to routine non-invasive monitors, bilateral radial arterial lines and a right internal jugular vein 9 French multi access catheter were placed. TEE was routinely used. A pulmonary artery catheter (PAC) is not used routinely in our institution unless indicated by specific pathology. None of the patients in this series were monitored with a PAC.

### Case 1

A 54-year-old male, with alcohol-related liver cirrhosis and a calculated Model for End- stage Liver Disease (MELD) score of 28, presented for deceased donor LT. ESLD was complicated by hepatic encephalopathy, ascites, spontaneous bacterial peritonitis (SBP), and esophageal varices. A preoperative transthoracic echocardiogram (TTE) performed 10 months before transplantation demonstrated normal size and systolic function of both ventricles (RV and LV), no valvular or regional wall motion abnormalities, normal pulmonary artery pressures, and a left ventricular ejection fraction (EF) of 65%. The TTE did, however, demonstrate bi-atrial dilatation, and evidence of diastolic dysfunction with an E/A ratio of 0.9, a deceleration time (DT) of 278 ms, and tissue Doppler early diastolic velocities of 8 cm/s at the annulus and 12 cm/s at the septum indicating impaired relaxation. References for degree of diastolic dysfunction are provided in Table 1. A dobutamine stress echocardiogram (DSE) was negative for ischemia and an electrocardiogram (EKG) performed at the same time as TTE demonstrated normal sinus rhythm with a prolonged QTc interval of 476 ms.

**Table 1:** Doppler echocardiographic values for evaluation of left ventricular diastolic dysfunction

Parameter	Normal	Impaired relaxation	Pseudonormal filling	Restrictive filling
E/A (cm/s)	> 1	< 1	1–2	> 2
DT (ms)	< 220	> 220	150–200	< 150
IVRT(m/s)	< 100	> 100	60–100	< 60
S/D	≥ 1	≥ 1	< 1	< 1
E’(cm/s)	> 8	< 8	< 8	< 8

E/A: Early to late left ventricular (LV) filling ratio

DT: Early LV felling deceleration time

IVRT: Isovolumic relaxation time

S/D: systolic to diastolic pulmonary venous flow ratio

E’: peak early diastolic mitral annular velocity

Adapted after: [35]

Shortly after the beginning of pre-anhepatic phase, transesophageal echocardiography (TEE) demonstrated an EF of 40–45% with no wall motion abnormalities. The surgical procedure was complicated by blood loss of 5.5 l with the patient receiving 3 L of crystalloids, 1 L of 5% albumin, 16 units of fresh frozen plasma (FFP), 15 units of packed red blood cells (PRBC), 3 units of platelet concentrate, and 3 units of cryoprecipitate. Despite the significant blood loss and reduction in EF, hemodynamic stability was maintained throughout the case with minimal vasopressor support (norepinephrine (NE) infusion 0.02–0.05 mcg/kg/min with intermittent boluses (10–20 mcg) during reperfusion). Hemodynamics and arterial blood gas data is presented in Table 2.

**Table 2:** Hemodynamic and arterial blood gas data

	After Intubation	Prior to Clamping	Post Reperfusion	Prior to Transport to ICU
Patient 1				
MAP (mm Hg)	60	69	56	67
MAP mean (mm Hg)	65			
CVP (mm H_2_O)	20	20	12	7
CVP mean (mm H_2_O)	11.8			
FiO_2_	0.5	0.5	0.5	0.5
PaO_2_ (mm Hg)	234	73	170	138
pH	7.33	7.35	7.35	7.41
BE	−2	-2	−4	0
Patient 2				
MAP (mm Hg)	64	84	70	85
MAP mean (mm Hg)	77			
CVP (mm H_2_O)	11	13	9	5
CVP mean (mm H_2_O)	9.6			
FiO_2_	0.5	0.5	0.5	0.5
PaO_2_ (mm Hg)	160	160	198	184
pH	7.512	7.424	7.33	7.31
BE	9	8	0	0
Patient 3				
MAP (mm Hg)	60	58	71	71
MAP mean (mm Hg)	68			
CVP (mm H_2_O)	14	12	16	18
CVP mean (mm H_2_O)	15			
FiO_2_	0.6	0.6	0.6	0.6
PaO_2_ (mm Hg)	100	124	216	210
pH	7.36	7.36	7.29	7.34
BE	−3	−3	−5	−2

*MAP* Mean Arterial Pressure, *CVP* Central Venous Pressure, *FiO*_*2*_ Fraction of Inspired Oxygen, *PaO*_*2*_ Partial Arterial Oxygen Pressure, *BE* Base Excess

The patient was admitted to the Surgical Intensive Care Unit (SICU) for postoperative management in stable condition.

In the SICU, the patient initially remained intubated and sedated with propofol and fentanyl infusions titrated to a Riker Sedation Score of 3–4. Weaning attempts failed due to episodes of agitation and hypertension. Despite a normal postoperative hepatic Doppler study and both laboratory and clinical improvement in liver function, the patient’s neurological status did not improve. A brain MRI performed on post-operative day (POD) 3 was normal. On POD 5, the patient’s mental status improved significantly and he was successfully extubated. A few hours after extubation, however, the patient complained of difficulty breathing and became hypoxic with chest X-ray findings consistent with acute pulmonary edema. This episode resolved with aggressive diuresis and continuous positive airway pressure (CPAP). On POD 6, a similar episode occurred but was only minimally responsive to escalating doses of diuretics and CPAP.

TTE performed at that time demonstrated diffuse LV hypokinesis, an EF of 25%, dilated left and right atria, and a dilated RV with globally reduced function. EKG demonstrated a prolonged QTc of 510 ms with no new ST-T changes. Three sets of cardiac enzymes performed 4 h apart were negative. Later that day, the patient developed new onset atrial flutter with episodic arterial desaturation requiring re-intubation and mechanical ventilation. A pulmonary artery catheter (PAC) was placed and a dobutamine infusion was started with a goal to keep the mean arterial pressure (MAP) above 65 mmHg (PAC data in presented in Table 3). Over the next few days, the patient developed progressively worsening hypotension, requiring escalating doses of vasopressors. Daily TTE demonstrated continuing deterioration of cardiac function with an EF as low as 10%. The patient’s renal function deteriorated and continuous renal replacement therapy (CRRT) was begun.

**Table 3:** Pulmonary artery catheter data for Patient 1

Time of measurement	CI L/min/m^2^	PAP s/d/(m) mmHg	CVP cmH_2_O	PCWP mmHg	Vasopressor requirement
POD 7 (initial)	1.6	29/21/(23)	16	–	Dobutamine 5mcg/kg/min
POD 9 (day before ECMO)	2.0	38/26/(29)	17	–	Dobutamine 7.5mcg/kg/min Vasopressin 0.03mcg/kg/min
POD 22 (on VA ECMO after Impella removal)	3.5	21/15/(17)	6	15	Epinephrine 0.2mcg/kg/min Norepinephrine 5mcg/kg/min Vasopressin 0.04mcg/kg/min Milrinone 0.6255mcg/kg/min
POD 29	4.0	55/33/(37)	14	29	Vasopressin 0.1u/kg/min Norepinephrine 0.8 mcg/kg/min Epinephrine 0.04 mcg/kg/min

*CI* Cardiac index, *PAP* Pulmonary Artery Pressure, *CVP* Central Venous Pressure, *PCWP* Pulmonary Capillary Wedge Pressure

Due to worsening cardiogenic shock, the patient was placed on veno-arterial extracorporeal membrane oxygenation (VA-ECMO) on POD 10. Despite ECMO support, the LV remained distended and globally hypokinetic. An Impella® device (Abiomed, Danvers, MA, USA) was placed to provide ventricular decompression. Over the course of the next few days, inotropic support was weaned and TTE demonstrated decreased LV dilation and an improvement in function (EF of 40%). The Impella® device was discontinued and a low dose epinephrine infusion was started. The patient tolerated a clamp trial and VA-ECMO was weaned. At the same time, however, liver transaminases began to increase. Liver Doppler evaluation demonstrated thrombosis of the left portal vein and decreased flow in the hepatic arteries. This was despite being maintained on a heparin infusion with a target activated partial thromboplastin time (aPTT) of 50–60 s. His clinical condition continued to deteriorate requiring an escalation of vasopressor support.

Postoperatively, this patient received 20 U PRBC (not more than 2 U/day), 1 U of FFP (on POD 0), and 8 U of platelets. Crystalloids were used as maintenance and fluid management was directed using TTE. After extensive discussions with the family, care was withdrawn and the patient expired on POD 31.

### Case 2

A 47-year-old male, with alcohol-related liver cirrhosis and a calculated MELD score of 39, presented for deceased donor LT. His ESLD was complicated by esophageal varices, upper gastrointestinal bleeding, and SBP. This patient’s abnormal laboratory studies included a serum iron level of 144 mg/dl (normal range 49–181 mg/dl), ferritin of 3670 ng/ml (normal range 17.9–464 ng/ml), and iron saturation of 85% (normal range 20–55%). As a result of these abnormal lab results, genetic testing was performed to determine if there was any genetic predisposition to hemochromatosis. Genetic testing did, in fact, reveal that the patient was heterozygous for HFE (HFE-H63D) and alpha-1 antitrypsin (PiSZ), predisposing him for hemochromatosis. Preoperative TTE performed 2 months prior to LT demonstrated mild left ventricular hypertrophy with an EF of 55%, mild bi-atrial dilatation, and a dilated RV with normal systolic function. There were no valvular abnormalities and pulmonary arterial pressures were normal. In addition, the TTE demonstrated some degree of diastolic dysfunction (impaired relaxation) with an E/A ratio of 1.1, a DT of 228 ms, and tissue Doppler early diastolic velocities of 6 cm/s at the annulus and 9 cm/s at the septum. EKG demonstrated a prolonged QTc of 479 ms. Myocardial Perfusion Scintigraphy (MPS) performed 3 weeks before LT demonstrated an EF of 54% with no evidence of ischemia or infarction.

The surgical course was uneventful with an estimated blood loss of 1.6 l. Intraoperatively, the patient received 5 units PRBC, 2 units of platelet concentrate, 1 L blood from cell saver, 2000 mg of fibrinogen (RiaSTAP), 1000 units of prothrombin complex concentrate (Kcentra), and 1 L of crystalloid. Intraoperatively, this patient required NE administration (0.02–0.08 mcg/kg/min with 0.2 mcg/kg/min for a short period of time during the anhepatic phase). Hemodynamics and arterial blood gas results are presented in Table 2. Intraoperative TEE demonstrated normal cardiac function with an EF of 55% and no valvular abnormalities. The patient was admitted to the SICU for postoperative management where he was extubated on POD 1.

On POD 4, the patient’s mental status decreased significantly and had an increasing oxygen requirement. Auscultation of the lungs demonstrated wheezing in all lung fields and chest X-ray findings were consistent with acute pulmonary edema. TTE at that time demonstrated a mildly dilated LV with diffuse hypokinesis and severely reduced systolic function (EF 20%). The left atrium was severely dilated and there was a moderate degree of mitral regurgitation. The RV was severely dilated and diffusely hypokinetic with reduced systolic function. Troponins were mildly elevated (0.024 ng/ml (normal< 0.010 ng/ml)) and brain natriuretic peptide (BNP) was significantly elevated (7625 pg/ml (normal < 125 pg/ml)). EKG demonstrated sinus tachycardia with no ST-T changes. The patient received aggressive diuresis along with CPAP. A beta-blocker and heparin infusion was started. Coronary angiography was performed which was unremarkable.

Over the next few days, the patient’s cardiac function remained unchanged (as assessed by serial bedside TTEs), however, his hemodynamics continued to deteriorate. A PAC was placed and dobutamine and NE infusions started with a goal to keep the MAPs above 65 mmHg (please see PAC data in Table 4). After the administration of catecholamines, the patient continued to deteriorate and was re-intubated. He also developed a severe metabolic acidosis, supraventricular arrhythmias requiring cardioversion, and acute renal failure requiring CRRT. His liver function continued to deteriorate and he became progressively encephalopathic. An abdominal CT scan demonstrated a hepatic artery thrombosis. A heparin infusion was started and titrated to target an aPTT of 50–70 s. In view of a continued decline in cardiac and hepatic function, the decision was made to withdraw care on POD 22.

**Table 4:** Pulmonary artery catheter data for Patient 2

Time of measurement	CI L/min/m^2^	PAP s/d/(m) mmHg	CVP cmH_2_O	PCWP mmHg	Vasopressor requirement
POD 13 (initial)	1.6	53/43/(48)	–	16	Dobutamine-5mcg/kg/min Norepinephrine-0.18mcg/kg/min
POD 16	1.8	38/26/(29)	28	–	Dobutamine 5mcg/kg/min Norepinephrine 0.2mcg/kg/min
POD 19	1.8	31/23/(25)	16	–	Dobutamine 5mcg/kg/min Norepinephrine 0.2mcg/kg/min Milrinone 0.625 mcg/kg/min
POD 22	1.4	34/18/(30)	17	–	Epinephrine 0.2 mcg/kg/min Vasopressin 0.04 mcg/kg/min Norepinephrine 0.5 mcg/kg/min Milrinone 0.625 mcg/kg/min

*CI* Cardiac index, *PAP* Pulmonary Artery Pressure, *CVP* Central Venous Pressure, *PCWP* Pulmonary Capillary Wedge Pressure

Postoperatively, this patient received 5 U of PRBC and 2 U platelets. Cardiac function was evaluated by daily TTE performed by a certified ICU physician. Fluid administration was also directed by TTE.

Microscopic examination of the explanted liver demonstrated signs of alpha-1 antitrypsin deficiency (globules within hepatocytes) as well as very large amounts of iron deposits within the hepatocytes; a sign of hereditary hemochromatosis. Postmortem pathology demonstrated an enlarged heart (490 g), RV hypertrophy, dilation of all valves, and minimal atherosclerotic changes of the left main coronary artery, left anterior descending artery, and aorta. Microscopic examination of cardiac tissue demonstrated stainable iron within both the myocytes and cells of the conduction system.

### Case 3

A 64-year-old male patient, with cryptogenic liver cirrhosis and hepatocellular carcinoma with calculated MELD score of 21, presented for a deceased donor LT. His ESLD was complicated by recurrent ascites, non-bleeding esophageal varices, portal hypertensive gastropathy, and hepatic hydrothorax. His other medical problems included a prior myocardial infarction (3 years prior to LT) treated with a bare metal stent, Grave’s disease, and asthma. A TTE performed 3 months prior to LT revealed a small LV cavity with normal systolic function (EF of 63%), no valvular or regional wall motion abnormalities, a small pericardial effusion, and normal pulmonary artery pressures. The E/A ratio in this case was 0.74 with a deceleration time of 289 ms. Tissue Doppler early diastolic velocities were 8 cm/s at the annulus and 11 cm/s at the septum indicating impaired relaxation. Preoperative EKG demonstrated a prolonged QTc of 467 ms. MPS performed 3 months before LT demonstrated an unchanged fixed deficit in the infero-lateral wall.

The patient’s surgical course was complicated by blood loss of 5 l, primarily during the pre-anhepatic stage due to significant adhesions from repeated paracentesis. He received 24 units of PRBC, 24 units of FFP, 3 units of platelet concentrate, 1000 mg of fibrinogen (RiaSTAP), and 4.5 L of crystalloid. Despite the significant blood loss, hemodynamics was maintained within a normal range with minimal vasopressor support (NE was administered 0.03–0.7 mcg/kg/min with 0.3 mcg/kg/min for a short period of time during the anhepatic phase). Hemodynamics and arterial blood gas analysis are presented in Table 2. Intraoperative TEE demonstrated an EF of 65%. The patient was admitted to the SICU for postoperative management and was extubated on POD 1.

On POD 2, he developed acute respiratory distress with hypoxemia (SpO2 < 90%) and an increasing oxygen requirement. Chest x-ray demonstrated acute pulmonary edema with bilateral moderately sized pleural effusions. TTE at this time revealed a mildly dilated left ventricle with severely reduced systolic function (EF of 20%) and diffuse hypokinesia. The RV was also dilated with reduced systolic function. Pulmonary artery pressures were mildly elevated and there was no valvular dysfunction. Other laboratory results obtained on the same day demonstrated an elevated troponin (0.182 ng/ml) as well as a markedly increased BNP (greater than 35,000 pg/ml). EKG demonstrated normal sinus rhythm with a prolonged QT of 488 ms. Troponins peaked at 0.463 on POD 3 and then trended down. With aggressive diuresis and ventilator support, the episode resolved. Repeat TTE performed 3 days later demonstrated improving LV systolic function (EF of 40%) and normalization of pulmonary artery pressures. The patient was discharged from intensive care on POD 8, liver enzymes normalized by POD 13, and LV size and function returned to normal (EF 55%) on POD 32. Postoperatively, this patient received 1 U FFP on POD 6. TTE was routinely performed to assess cardiac function and direct fluid administration.

## Discussion

We have described 3 cases of acute HF after LT presenting in the immediate postoperative period. Preoperative cardiac evaluation was normal in all patients and, with the exception of moderately large intraoperative blood loss, each patient’s intraoperative course was uneventful. One patient had a mildly reduced EF intraoperatively, however, neither this patient nor the other two had any significant hemodynamic alterations during surgery. Post-operatively, all three patients developed acute HF associated with biventricular dysfunction and dramatic decreases in EF to about 20%. Two of the three patients progressed to cardiogenic shock with multi-organ systemic failure and expired after maximal therapy. Only one patient survived the acute episode.

These cases highlight the possibility that an unknown, underlying cardiac condition was not identified by the standard cardiac evaluation recommended for LT candidates.

Despite US transplantation centers following the guidelines for preoperative cardiac evaluation recommended by the American Association for the Study of Liver Disease (AASLD) [2], up to 21% of all deaths after LT are related to cardiac failure [3]. The overall incidence of post-transplant HF has been reported to be as high as 24% [4–6], with a reported mortality of up to 45% [1, 7]. Aside from negatively affecting a patient’s overall prognosis, impaired cardiac function after LT is also associated with the development of additional complications including acute kidney injury, life-threatening arrhythmias, infections, and graft failure [8].

The etiology of acute HF after LT is not well understood. It is likely that HF presenting in the immediate postoperative period, and HF remote from transplantation, have different origins.

The cause of HF in the immediate post-operative period is likely multifactorial and can be directly related to the patient, the graft, and surgical factors. Intraoperative management can also affect postoperative outcome.

ESLD itself is associated with significant changes in the physiology of almost all organ systems. Patients with liver failure are prone to hemodynamic instability due to profound vasodilatation related to both endotoxin release and nitric oxide dysregulation [9, 10]. Despite these intrinsic handicaps, LT candidates usually present well compensated and with stable preoperative hemodynamics, however, they may decompensate. Intraoperatively, however, these patients frequently become hemodynamically unstable. LT itself is associated with acute changes in preload as well as the release of cytokines and toxins that can lead to acute decompensation in the form of post-reperfusion or/and vasoplegic syndromes [11–15].

None of the patients in our series had any preoperative findings that would have excluded them from transplantation. High quality grafts were used for all three cases and the surgical procedure was, in general, uncomplicated. There was no profound intraoperative hemodynamic instability. TEE was used to optimize intraoperative fluid management; in particular, to avoid fluid overload.

While the exact cause of post-operative HF might be unknown, there are a number of causes of ESLD that are primarily associated with cardiac dysfunction and can be broadly classified into ischemic and non-ischemic.

Ischemic causes of acute HF are relatively uncommon in patients with ESLD. Because of the well-known association between coronary artery disease (CAD) and high mortality rates in ESLD [16], a standard cardiac evaluation usually identifies patients with significant coronary problems. A recent large multi-center retrospective study performed by Wray, et al. has demonstrated that LT performed in patients with CAD have better outcomes than previously estimated [17]. In our group of patients, an ischemic etiology of HF was ruled out. Preoperative CAD testing was performed in all patients and was negative. Patient 1 did not have any EKG changes or troponin elevation, while patient 2 underwent cardiac catheterization which did not show any signs of CAD. Patient 3 had a history of ischemic heart disease with an area of fixed defect on MPS but only developed a small enzyme leak, with no EKG changes suggestive of ischemia, during the acute episode.

Non-ischemic causes include cardiomyopathy related to hemochromatosis, alcohol abuse, sepsis, cirrhotic cardiomyopathy (CCM), non-alcoholic steatohepatitis (NASH) and stress-induced (Takotsubo) cardiomyopathy [18, 19]. Takotsubo cardiomyopathy is a rare condition that can mimic acute coronary syndrome. It has been previously described in patients undergoing LT [20]. This disorder can be associated with significant hemodynamic instability, QTc interval prolongation, ST segment elevation or depression, T-wave changes, and increases in cardiac enzymes [21]. Takotsubo cardiomyopathy is associated with a typical echocardiographic presentation that includes akinesis of the apical and distal anterior wall in combination with hyperkinesis of the basal wall [22]. None of our patients had echocardiographic features consistent with this type of stress-induced cardiomyopathy.

Our patients did not demonstrate any clinical, laboratory or radiologic signs of sepsis or systemic inflammatory response syndrome.

HF can be the result of fluid overload. One of the reasons to use TEE for LT is to optimize fluid management. Liver congestion due to fluid overload can lead to increases in portal pressure which result in graft dysfunction and coagulopathy. Transplanted graft also can be affected by over-transfusion. Transfusion Related Lung Injury (TRALI) and Transfusion Associated Circulatory Overload (TACO) have been described in LT recipients [23]. We used routinely TEE/TTE for the management of all our cases and none of our patients had any signs of TACO or TRALI. In addition, there was no indication of fluid overload at any time during the intra- or postoperative management. Patients with these findings must be managed very carefully with special attention to avoid fluid overload. We believe the best way to achieve this is with the routine use of TEE monitoring.

All three patients, however, had mild diastolic dysfunction as manifested by a low E/A ratio and prolonged deceleration time on their preoperative echocardiogram. In each patient, there was preoperative prolongation of the QTc interval, which increased significantly during the acute episode. Both diastolic dysfunction and prolonged QTc intervals > 450 ms have been associated with the development of new-onset systolic heart failure after liver transplantation [6, 7]. The etiology of diastolic dysfunction varied between patients, with one patient having cardiomyopathy related to hemochromatosis, and the other two most likely having CCM.

Diagnosis of CCM is very challenging [24]. Standard stress imaging, such as DSE (recommended by AASLD) or MPS, is unable to fully evaluate the profound impact of cirrhosis on myocardial integrity and reserve. There are numerous reports of DSE not being able to accurately evaluate a blunted inotropic response to dobutamine in cirrhotic patients (one of the features of CCM), yielding low negative predictive values because of the inability to reach the target peak double product [25]. Cardiac MRI and sophisticated imaging methods such as strain and strain rate may identify subtle LV dysfunction [26]. It is not routine practice to perform these imaging techniques for all LT candidates, and the efficacy of this test in patients with CCM is unclear. Dowsley, et al. retrospectively demonstrated an association between diastolic dysfunction and early postoperative HF in LT recipients [4]. Other additional diagnostic indicators of CCM include increased BNP levels to 400 pg/ml and above [27], and elevations in high sensitivity troponin T [28]. Although various retrospective studies have identified these features as risk factors for development of HF after LT, there is no consensus on what next steps should be taken with either preoperative assessment or intraoperative management.

For patients with iron overload cardiomyopathy, cardiac magnetic resonance imaging (CMRI)-derived T2 relaxation time is currently the mainstay for quantitative assessment of myocardial iron deposition [29]. The elevated preoperative ferritin levels in one of our patients was felt to be due to the large number of blood transfusions he received for variceal bleeding. Because of this, a CMRI was not performed. Iron chelation therapy has been effective in preventing the development of LV dysfunction and heart failure in patients with iron overload [30]. It is unclear if chelation therapy would have been helpful in our patient if myocardial iron deposits had been identified before transplantation.

We recognize that not having a cardiac evaluation immediately before transplantation is a significant limitation in this case series. The AASLD recommends an annual cardiac ultrasound for liver transplant candidates. Although the majority of LT centers in the US base their guidelines for preoperative evaluation on the AASLD recommendations, the timing of the preoperative cardiac ultrasound varies significantly. Unless a patient has a particular indication to perform this examination more frequently, it should be performed yearly. In our case series, a TTE was performed between 2 and 10 months before transplant. When the high mortality associated with HF in patients undergoing LT is taken into consideration, the current guidelines should be revised to include a TTE evaluation close to the time of transplant. The treatment for non-ischemic cardiomyopathy developing after LT is symptom based. This includes inotropic support, cautious use of diuretics, and careful management of preload and afterload [31, 32]. In patients with worsening organ function and delayed cardiac recovery, early institution of advanced hemodynamic support techniques such as ECMO may be valuable options. It has been demonstrated that cardiac dysfunction after LT, in most patients with cirrhosis, is reversible with full recovery of functional, structural, and electrophysiological abnormalities and normalization of liver function [33, 34]. Although one of our patients recovered cardiac function upon institution of ECMO support, he succumbed to acute graft failure due to hepatic artery thrombosis. Further data is needed before ECMO can be routinely recommended in these patients.

## Conclusions

We have presented three patients who underwent LT and developed HF in the immediate post-operative period. They all underwent a complex preoperative evaluation and an uneventful transplantation, and were hemodynamically stable throughout surgery. Postoperatively, all three patients developed acute HF associated with dilated cardiomyopathy. Only one patient recovered from the acute episode. Although risk factors for development of acute HF after LT have been identified in retrospective studies, there is still a lack of clarity concerning optimal evaluation and management. Questions regarding the threshold for ordering specialized cardiac studies and the efficacy of these studies, as well as withholding candidacy for LT in these patients, remain unanswered.

Based on our current knowledge, the combination of diastolic dysfunction, QTc interval prolongation (above 450 ms), and an increased BNP level (above 400 pg/ml) are predictive of HF in the postoperative period. Patients with these findings must be managed very carefully with special attention to avoid fluid overload. We believe the best way to achieve this is with the routine use of TEE monitoring. Once acute HF has occurred, multidisciplinary management is recommended. In patients with delayed cardiac recovery and worsening organ function, the use of advanced hemodynamic support techniques (ECMO) is controversial and needs further research.

## Abbreviations

(NASH): non-alcoholic steatohepatitis
AASLD: American Association for Study of Liver Disease
aPTT: Activated Partial Thromboplastin Time
BNP: Brain Natriuretic Peptide
CAD: Coronary Artery Disease
CCM: Cirrhotic Cardiomyopathy
CMRI: Cardiac Magnetic Resonance Imaging
CPAP: Continuous Positive Airway Pressure
CRRT: Continuous Renal Replacement Therapy
DSE: Dobutamine Stress Echocardiography
DT: Deceleration Time
E/A: Early to late left ventricular filling ratio
EF: Ejection Fraction
EKG: Electrocardiogram
ESLD: End Stage Liver Disease
FFP: Fresh Frozen Plasma
HF: Heart Failure
LT: Liver Transplantation
LV: Left Ventricle
MAP: Mean Arterial Pressure
MELD: Model for End Stage Liver Disease
MPS: Myocardial Perfusion Scintigraphy
NE: norepinephrine
PAC: Pulmonary Artery Catheter
POD: Postoperative Day
PRBC: Packed Red Blood Cells
RV: Right Ventricle
SBP: Subacute Bacterial Peritonitis
SICU: Surgical Intensive Care Unit
TACO: Transfusion Associated Circulatory Overload
TEE: Transesophageal echocardiogram
TRALI: Transfusion Related Lung Injury
TTE: Transthoracic Echocardiogram
VA-ECMO: Veno-arterial Extra Corporeal Membrane Oxygenator
